# Help From and Help to Neighbors Among Older Adults in Europe

**DOI:** 10.3389/fsoc.2019.00046

**Published:** 2019-05-30

**Authors:** Alexander Seifert, Ronny König

**Affiliations:** ^1^University Research Priority Program “Dynamics of Healthy Aging”, University of Zurich, Zurich, Switzerland; ^2^Institute of Sociology, University of Zurich, Zurich, Switzerland

**Keywords:** given neighborhood help, received neighborhood help, Europe, older adults, SHARE

## Abstract

Neighborhoods can be a valuable source of social support particularly for older adults. Such support can be mutual; however, the influential factors in giving and receiving are unclear. This study investigated neighborhood help among the older European population using representative data for 17 countries from the Survey of Health, Ageing and Retirement in Europe (SHARE). The analyses were based on 104,059 observations of 39,113 respondents aged 50 years and older. In general, ~6% of all respondents provided recently neighborhood help, and 4% received help. Moreover, the results indicate a high degree of reciprocity in giving and receiving neighborhood help. However, the situation varied widely among age groups and countries. Our multilevel results suggest that the provision and receipt of help are driven by personal characteristics (age, sex, education, income, and retirement), health resources (subjective health, activities of daily living, and instrumental activities of daily living), living situation (homeownership, location, and length of time in a residence), social factors (marital status, partner, parents, and children), and contextual factors (gross domestic product, social expenditures, poverty rate, Gini index, population density, country-centered satisfaction with life, living, and relationships).

## Introduction

People live in neighborhoods, and their interactions with their neighbors are shaped by social contacts and support. Hamm (1973) defined neighbors as social groups whose members interact primarily because of the commonality of the place of residence. In this sense, neighbors are by definition nearby. Mutual support is not necessarily provided; however, neighborhoods are community-building locations characterized by social, functional, cultural, or circumstantial connections (Chaskin, 1997). Thus, the neighborhood has proved to be an arena that is suitable for social exchange due to the factors of proximity, continuity, and place attachment, which promote the development of social contacts (Henning and Lieberg, 1996; Oswald et al., 2005;Burns et al., 2012).

### Help Among Neighbors

According to Keller (1968), neighborhoods can be divided conceptually into the following elements: neighbor, neighboring, and neighborhood. The term “neighbor” defines a person's role and the attitudes, expectations, and negotiations deriving from the resulting interactions, whereas “neighboring” refers to the social activities pursued by neighbors, and the territorial term “neighborhood” describes the spatial area that is physically and symbolically different from the greater environment.

This study focused exclusively on the role of neighboring, which Keller (1968) defined as “activities engaged in by neighbors as neighbors and the relationships these engender among them” (p. 29). The core component of the concept of neighboring is the considerations of contact and help exchange within a given neighborhood (Farrell et al., 2004; Kusenbach, 2006). The present study focused on informal neighborhood help that represents the private contact and help—not organized through third parties—that can be found in a neighborhood. Help exchange can occur in different forms, for example, emotional or instrumental support (Unger and Wandersman, 1985). Because neighborly assistance is not unidirectional, the mutual dimension was considered, and a distinction was made between given help, i.e., any help provided to neighbors, and received help, i.e., any help from neighbors. Social exchange theory assumes that people tend to maintain a balance among support exchanges. This is known as the norm of reciprocity (Gouldner, 1960; Mauss, 2000): Individuals prefer relationships in which they receive and give a more or less equal amount of support (Antonucci and Jackson, 1990). The less the support that is exchanged, the greater is the chance that relationships will end or become peripheral (e.g., van Tilburg, 1992; Ikkink and Van Tilburg, 1999).

In their convoy model of social relations, Kahn and Antonucci (1980) proposed that from childhood to old age, individuals are surrounded by several people with whom they regularly interact and exchange instrumental and emotional support (Antonucci et al., 2010). These social relationships tend to focus on fewer individuals and become more selective in old age (Conway et al., 2013). Older adults are also selective about when and from whom they seek support and the kind of support they seek. The convoys vary by personal, situational, and contextual characteristics and have important implications for well-being (Antonucci et al., 2010). In terms of importance, neighbors rank lower than family members and close friends (Sander et al., 2017). Nevertheless, if family members or friends do not live close by or are not readily available, neighbors may provide important needed support.

The exchange of help among neighbors can have an effect on the personal support network that helps individuals to cope with everyday life (Oswald et al., 2011; Murayama et al., 2015). Hoogerbrugge and Burger (2018) asserted that neighborhood-based support is important mainly for individuals, particularly those who are retired or are in poor health, who are more likely to spend a considerable amount of time in a neighborhood. Therefore, older adults, mainly those who are retired and/or are in poor health, are a particularly important group for studying neighborhood help.

### Neighborhood Help Among Older Adults

According to the World Health Organization (WHO), most people in developed countries can expect to live at least into their 60s; consequently, the older population has been growing significantly (WHO, 2015). These demographic changes have profound implications for every society. The expectation is that the number of older people who are retired and are facing limitations in everyday life will increase, as will their reliance on their immediate neighborhoods. This issue becomes especially critical in times of declining fertility rates, smaller family sizes, and greater mobility. Thus, some individuals might have no family members, or their family might live at a great distance from them (Brandt et al., 2009; Isengard and Szydlik, 2012). Whereas help from family members might be limited, support from friends or neighbors is more accessible and thereby becomes more important (Boerner et al., 2016; Deindl and Brandt, 2017).

Therefore, it is not surprising that neighboring has been widely discussed in the context of older adults (e.g., Lawton and Brody, 1969; Schwirian and Schwirian, 1993; Oswald and Wahl, 2004). According to Cantor (1979), the domestic environment becomes more important in old age, primarily because of personal limitations (e.g., health, mobility, and social networks) and place attachment (see also the work of Lawton and Nahemow, 1973; Shaw, 2005; Wahl et al., 2012). Older adults spend a great deal of time in their homes and neighborhoods. Shaw (2005) demonstrated that the expectation of support from neighbors is stronger among older than younger adults primarily because older adults have more frequent contact with neighbors and higher residential stability (Glass and Balfour, 2003;Heinze et al., 2015).

Having ties to neighbors facilitates access to informal aid and reduces the sense of isolation, which can mitigate the problems of maintaining everyday life at a time of advancing age and declining health (Beard et al., 2009; Yen et al., 2009; Oswald et al., 2011; Kalwij et al., 2014; Boerner et al., 2016; Ward et al., 2018). Such challenges include accessing home care (e.g., Nocon and Pearson, 2000; Barker, 2002; Kalwij et al., 2014; Pleschberger and Wosko, 2017), managing depressive symptoms (e.g., Aneshensel et al., 2007; Wight et al., 2009; Wang et al., 2018), maintaining physical functioning (e.g., Balfour and Kaplan, 2002), and achieving other wellbeing outcomes (e.g., Wahl et al., 2009; Morita et al., 2010; Stroope et al., 2017; Gallardo-Peralta et al., 2018). Therefore, older adults' experiences and behaviors in terms of wellbeing, independence, and social integration are closely related to their environments: their neighborhoods (Wahl et al., 2012).

### The Missing Link: Factors Influencing Given or Received Neighborhood Help

Neighborhood help, particularly its role in facilitating older adults' everyday life, has been studied extensively; however, there is a lack of research regarding the predictors of help given to and received from neighbors. The convoy model (Kahn and Antonucci, 1980) suggests that social convoys change with age, with neighbors becoming more important in advanced age, but this can vary according to personal and situational characteristics (Antonucci et al., 2010). The description of these personal and situational characteristics is scarce in previous literature. Therefore, it is crucial to investigate the determinates of given and received neighborhood help among older adults. Despite reductions in public services and the general view of neighbors as a potential source of informal support for older adults, few studies have documented older individuals' perspectives on receiving help from and, particularly, giving help to neighbors (Grime, 2018). It is, therefore, important to assess the factors affecting neighboring, herein defined as given and received neighborhood help. These factors should be examined at the micro-, meso-, and macrolevels.

At the microlevel, personal characteristics that can influence the willingness to provide or the need to receive neighborhood help include age, gender, education, income, and health status. Studies have, for example, demonstrated that the incidence of home care, such as help from neighbors, is highest for the oldest-old, for whom low mobility and other limitations impede the accomplishment of daily living activities (Shaw, 2005; Kalwij et al., 2014), and women are more likely to provide neighborhood assistance (Seifert, 2016). However, the interrelationships among personal education, income, and health status, and the effects of these factors on sensitivity and reciprocity concerning neighborhood help have received little attention. Furthermore, discussions on neighboring should consider the effect of the time spent living in a neighborhood on the help that is given and received.

Each individual lives in a social context; therefore, the mesolevel, defined as the individual's social environment, must also be taken into account. An important aspect of this social context is the presence of a partner. Findings underscore the importance of the partner as the main provider of social support, particularly for men (Weissman and Russell, 2018). However, the presence of this pattern of neighborhood help is unclear among older adults, who often face the loss of their loved one. A second source of family support is offspring; studies have demonstrated that elderly individuals who are childless or have children living in a different city or country generally receive less social support and have a greater reliance on formal support and care (Boerner et al., 2016; Deindl and Brandt, 2017). However, little is known about the role of children in the provision of neighborhood help, including the importance of co-residence or geographical distance. Besides a source of social and emotional support, having a family usually involves the fulfillment of social responsibilities, which not only include the education of young children, but also the care and support of aging parents, given the increase in life expectancy (Brandt et al., 2009). These responsibilities can result in time constraints, which in turn hinder the ability to help neighbors while simultaneously increasing the need for their emotional support. A review of the literature suggests that these relationships among older adults have not yet been thoroughly investigated.

Neighboring can also be influenced by the context in which a person lives, which includes the community or region as well as the culture and infrastructure. First, the regional context, e.g., a large city or rural area, can impact the assistance provided by neighbors. Second, the wealth and prosperity of a country can create positive or negative conditions for a lively neighborhood with a significant exchange of help among neighbors. Moreover, the country-specific infrastructure and conditions for social support, such as provisions for formal help and care, can create a situation in which families and neighbors are obliged to rely heavily on one another. A review of the literature suggests the country-specific factors impacting both given and received neighborhood help among older adults, but have yet not to be fully investigated. The present study therefore considered the contextual variations within and among 17 European countries in investigating the mechanisms behind the provision and receipt of neighborhood help.

In sum, the micro-, meso-, or macrolevel predictors of neighboring in old age are not yet well known. Few studies have focused on this important issue, and the limited results are conflicting. Most studies have explored the receipt of help from neighbors; however, this is merely one dimension of neighboring in general and for older adults in particular. The current study will therefore focus on the micro-, meso-, and macrolevel predictors of both given and received neighborhood help.

### Research Questions

On the basis of the above review, four research questions were posed:

1. How many older individuals offer and receive neighborhood help?
2. Is neighborhood help reciprocal within the older population?
3. Are age-related and country-specific variations of given or received neighborhood help observable?
4. Which factors on the micro-, meso-, and macrolevels influence neighborhood help?

Whereas, the first and second research questions focused on the mutual provision of neighborhood help among the older participants, the third question allowed for a detailed examination of age-related and country-specific variations in neighborhood help. Finally, the fourth research question considered the micro-, meso-, and macrolevel influences on given and received neighborhood help. Regarding personal characteristics at the microlevel, previous findings suggest that women and younger individuals with higher socioeconomic resources and good health would be more willing and able to support their neighbors. In contrast, older males with restricted socioeconomic resources and poor health would be more likely to receive help from neighbors. We also hypothesized that the length of time lived in the same home, and thus in the same neighborhood, is positively associated with both giving and receiving of neighborhood help.

The mesolevel factors examined in the study comprised the characteristics of the individual's social environment. We therefore hypothesized that respondents who were living in partnerships or had living parents and/or children residing nearby would be less involved in neighborhood help networks and therefore less likely to provide help to or receive help from neighbors.

In addition to micro- and mesolevel influences, the study examined some macrolevel indicators considered critical to explaining the differences in neighborhood help within and among the European countries that participated. First, it was hypothesized that older adults in rural areas would be more likely to give and receive neighborhood help than individuals in urban areas. Second, the same will be the case for wealthier and more generous countries with a high level of life satisfaction.

## Data and Methods

### Data

The analyses were based on the pooled data from the Survey of Health, Ageing and Retirement (SHARE) [see Börsch-Supan et al. (2013) for methodological details], a dataset that provides access to standardized information on respondents aged 50 years and older from 17 European countries. Partners, including those younger than 50, were also surveyed if they lived in the same household and were willing to participate. The countries included in the study were Austria, Belgium, Croatia, the Czech Republic, Denmark, Estonia, France, Germany, Greece, Italy, Luxembourg, Poland, Portugal, Slovenia, Spain, Switzerland, and Sweden. The dataset included a wide range of topics, such as demographics, income, health, accommodation, education, occupation, behavior, social support, activities, and expectations.

We used an unbalanced panel of respondents who were surveyed at least twice in one of the available waves conducted in 2004–2005 (Wave 1), 2006–2007 (Wave 2), 2010–2011 (Wave 4), 2013 (Wave 5), and/or 2015 (Wave 6). SHARELIFE (Wave 3) differs from previous and subsequent waves because of its retrospective questionnaire. Consequently, those data were excluded from the sample [for details on the data used in the study, see (Börsch-Supan, 2018a,b,c,d,e)]. Due to the focus of this study on the provision and receipt of help within neighborhoods, the sample was further restricted to the so-called “family” respondents, as they answered only the questions measuring the dependent variables. Last, the total sample comprised 104,059 observations of 39,113 respondents living in one of the 17 European countries. The data were adjusted for this investigation and extensive consistency checks were conducted.

### Dependent Variables

The provision of time-related given help was based on the introductory question: “in the last 12 months, have you personally given any kind of help … to a family member from outside the household, a friend, or a neighbor?” Types of help could include emotional, practical, or administrative support. If they indicated providing such help, then the respondents were further asked to name the recipients and their relationships. As the data did not differentiate between several types of help across all waves, any kind of assistance that was given to respondents' neighbors was coded “1,” whereas a lack of such assistance was coded “0.” For respondents who were surveyed in Waves 4 and 6, the information regarding help given was additionally generated by identifying a neighbor as part of their social network and reporting specific forms of assistance within the previous 12 months.

The second dependent variable, help received from at least one neighbor, was similarly constructed. This variable was based on the question “thinking about the last 12 months, has any family member from outside the household, or any friend or neighbor given you any kind of help…?” The dependent variable was coded “1” if the respondents answered “yes” and identified a neighbor in the follow-up question as the respective giver. The same information was used from the social network module that was available for Waves 4 and 6. Respondents who did not receive any kind of help from their neighbors within the previous 12 months were coded “0.”

### Independent Variables

To explain neighborhood help within the older population, several explanatory variables at the micro-, meso-, and macrolevels were considered. First, the interplay of given and received help among neighbors was measured by the inclusion of the respective other dependent variable as an explanatory variable in the models. In addition, the overall support network for each respondent was addressed through two variables, thereby allowing for the indication of “1” to represent the respondent's receipt of assistance from or provision of assistance to someone except a neighbor within the previous 12 months. The absence of given or received help was indicated by “0.”

For each respondent, gender (0 = female, 1 = male), age (continuous, in years), and socioeconomic status were considered. Socioeconomic status was indicated by respondent's highest educational level, which was recoded according to the International Standard Classification of Education (ISCED) in three tiers: low, ISCED 0–2; medium, ISCED 3–4; and high, ISCED 5 and higher. A subjective income measure was used to capture the financial situation of the entire household based on a question regarding the household's ability to meet expenses. Four response options were available: 1 = with great difficulty; 2 = with difficulty; 3 = fairly easily; and 4 = easily. The information regarding the respondents' status as homeowners (“1”) or not (“0”) was included as an indirect measurement of household wealth, and the length of time (in years) in a residence was used as an indicator of the comfort with the neighborhood and the possibility of establishing and maintaining contact with neighbors. Data on the indirect time spent providing assistance or the health restrictions affecting the ability to provide assistance were obtained by asking whether the respondent was retired (“1”) or not (“0”). Furthermore, the respondent's migrant status (0 = no, 1 = yes) was included.

Physical and mental health each play important roles in the probability of using neighborhood contacts and support (e.g., Kruger et al., 2007), and this information was obtained through health-related indicators. Objective health was recorded as the number of limitations on activities of daily living (ADL) and instrumental activities of daily living (IADL). Whereas, ADL include eating, dressing, personal hygiene, and walking, IADL encompass shopping, preparing meals, housekeeping, and managing finances. In addition, a subjective self-evaluation of the individual's health status was measured on a five-point scale (1 = excellent, 2 = very good, 3 = good, 4 = fair, 5 = poor).

To measure mesolevel influences, respondents' marital (1 = married, 2 = never married, 3 = divorced, 4 = widowed) and partnership statuses (1 = with a partner, 0 = without a partner) were included. To consider the closest family context, information on whether respondents had at least one living parent (0 = no, 1 = yes) was included, as was the geographical distance to their nearest child (ranging from 1 = same household or building to 6 =≥ 500 km). This information was coded “0” for childless respondents.

The regional background was included to address macrolevel differences related to culture and infrastructure within countries. The area of residence was based on five categories ranging from large cities (“1”) to villages or rural areas (“5”). Several macrolevel indicators representing the structural differences were considered in order to capture variations among the European countries that participated. Economic power and country-specific wealth were empirically expressed by the gross domestic product (GDP) per capita, adjusted for purchasing power and expressed in relation to the average of the 28 countries of the European Union (EU), set to 100. Public social and old age expenditures as a percentage of GDP were used as direct measures of state support. Thus, social expenditures represented the provision of benefits and contributions (e.g., cash transfers, goods and services) by public institutions to households and individuals. Retirement expenditures represented all public transfers for all types of pensions, such as (anticipated) old age, partial, disability, and survivors' pensions, and early retirement benefits for labor market reasons as well as reduced capacity to work.

Indirect consequences of state interventions were observable through the redistributive welfare-state policies. Accordingly, the models further measured the influence of poverty and income inequality (Gini coefficient). The poverty rate after taxes and transfers covered the proportion of the population for which income fell below 60% of the median household income (adjusted for household size with an equivalence scale) of the total population. The Gini coefficient indicated the extent to which the household income distribution in a country deviated from an equal distribution. Moreover, the national population density was included as a measure of the indirect availability of possible receivers and givers of help and defined as the ratio between the annual average population and the land area.

The influence of aggregated subjective measurements was tested by the inclusion of three personal wellbeing indicators for individuals 16 years and older who were living in private households. These measures included overall life satisfaction, which represented the individual's evaluation of the totality of his or her life, as well as satisfaction tested for two specific domains: living environment and personal relationships. The latter two detailed indicators also provided a broad subjective assessment of the specific area and individual situations and preferences at the national level.

Each of the aforementioned macro indicators was derived from official sources, such as the European Statistical Office (Eurostat) and the Organization for Economic Cooperation and Development (OECD), and refers to the year preceding each interview (see Table 1). To consider the possibility of variations in providing and receiving neighborhood help over time, the models were controlled for the wave related to each observation.

**Table 1:** Determinants of neighborhood help.

	**Help to neighbor**		**Help from neighbor**	
	**Gross**	**Net**	**Gross**	**Net**
	**OR**	**OR**	**OR**	**OR**
Help to neighbor			6.96^***^	7.45^***^
Help from neighbor	7.30^***^	7.75^***^		
Help to other	1.78^***^	1.69^***^	1.08	1.07
Help from other	1.21^***^	1.12	4.81^***^	3.56^***^
**MICROLEVEL**				
Male	1.25^***^	1.17^**^	0.74^***^	0.92
Age	0.89^***^	0.78^***^	1.43^***^	1.15^***^
**Education (Ref.: Low)**				
Medium	1.37^***^	1.15^***^	0.81^***^	1.13^**^
High	1.30^***^	1.07^*^	0.80^**^	1.22^***^
**Make Ends Meet (Ref.: With Great Difficulty)**				
With some difficulty	0.98	0.91	0.78^***^	0.86^*^
Easily	0.94	0.85^***^	0.65^***^	0.78^***^
Fairly easily	0.99	0.86^***^	0.66^***^	0.82^***^
Homeownership	1.01	0.86^***^	0.95	1.30^***^
Years lived in home	0.98	1.04^**^	1.22^***^	1.07^**^
Retired	1.17^***^	1.47^***^	1.44^***^	0.95^*^
Limitations (ADL)	0.85^***^	1.01	1.30^***^	1.06^**^
Limitations (IADL)	0.72^***^	0.73^***^	1.38^***^	1.08^***^
**Health Status (Ref.: Excellent)**				
Very good	0.84^***^	0.83^***^	1.04	1.04
Good	0.85^***^	0.85^***^	1.14	1.02
Fair	0.75^***^	0.80^***^	1.88^***^	1.42^***^
Poor	0.47^***^	0.55^***^	3.69^***^	2.15^***^
**MESOLEVEL**				
**Marital Status (Ref.: Married)**				
Never married	1.39^***^	0.95	2.48^***^	1.22
Divorced	1.14^**^	0.93^*^	1.92^***^	1.48^***^
Widowed	0.85^***^	0.84^***^	2.94^***^	1.67^***^
Partner	0.97	0.87^***^	0.41^***^	0.90
Parent(s)	0.81^***^	0.63^***^	0.61^***^	0.99
**Distance to Nearest Child (Ref.: Household/Building)**				
5 km	1.05	1.06^***^	1.47^***^	1.16
5–25 km	1.25^***^	1.17^***^	2.16^***^	1.80^***^
25–100 km	1.35^***^	1.25^***^	2.63^***^	2.28^***^
100–500 km	1.31^***^	1.18^**^	3.14^***^	2.99^***^
500 km/abroad	1.35^***^	1.31^***^	2.14^***^	2.19^***^
No children	1.57^***^	1.40^***^	3.47^***^	2.77^***^
Migrant	0.82^*^	0.93	0.83^*^	0.94
**MACROLEVEL**				
**Area (Ref.: Big City)**				
Suburbs	1.15	1.05	1.38^**^	1.45^***^
Large city	0.91	0.89	1.16	1.23^***^
Small city	0.98	0.94	1.23	1.35^***^
Village	1.10	1.04	1.39^**^	1.45^***^
GDP	1.21^***^	1.07^***^	0.95	1.14^***^
Social expenditures	0.97	1.14^***^	0.93^*^	1.15^***^
Old age expenditures	0.85^***^	0.99	0.99	1.14^***^
Poverty	0.91^**^	0.91^**^	0.89^***^	0.80^***^
Gini	0.86^***^	0.88^***^	0.76^***^	0.85^***^
Population density	1.07	1.38^***^	0.99	1.11^**^
Satisfaction, Life, 16+	0.98	1.22^***^	1.54^***^	1.39^***^
Satisfaction, Living, 16+	1.65^***^	1.12^**^	1.15^***^	1.28^***^
Satisfaction, Relations, 16+	0.48^***^	1.65^***^	1.09^**^	1.17^***^
*N* (observations)	104,059	104,059	104,059	104,059
*N* (respondents)	39,113	39,113	39,113	39,113
*N* (countries)	17	17	17	17

*** *p ≤ 0.01*,

** *p ≤ 0.05*,

* *p ≤ 0.10*.

*Data source: Survey of Health, Ageing and Retirement in Europe (SHARE), Waves 1, 2, 4, 5, and 6, Release 6.1.1; (Eurostat, 2018a,b,c,d,e,f), OECD (2018); multilevel logistic regressions (separate for each macro indicator and under control of the respective wave), robust standard errors, odds ratios (OR), own calculations*.

### Methods

In addition to providing a descriptive overview of neighborhood help in old age, the study aimed to explain the influences on such support through the results of multivariate analyses. Because of non-independence between observations, the hierarchical data structure of SHARE violated the basic regression assumptions for the unbalanced panel design and could result in inaccurate significance values and biased standard errors. To analyze the determinants of given and received neighborhood help, multilevel logistic regressions were performed at three levels: observations nested in respondents nested in countries using the statistical software package STATA with the Generalized Linear Latent And Mixed Model (GLLAMM) (see, e.g., Rabe-Hesketh and Skrondal, 2008; Snijders and Bosker, 2012; Hox et al., 2018). Variations at the upper level, i.e., the country level, were considered, as were those between respondents within and between countries. In addition to the fixed or released parameters at the observation and individual levels, these analyses considered the context variables separately to avoid the estimation biases that stem from multiple macrolevel indicators (Maas and Hox, 2005). All of the non-dichotomous variables were standardized to enable comparisons of the effects of the determinants at the micro-, meso-, and macrolevels. With the exception of the dependent variables, all of these variables had a mean value of 0 and a standard deviation of 1.

## Results

### Neighborhood Help Among Individuals Aged 50 and Older: A Descriptive Overview

**Figure 1:**
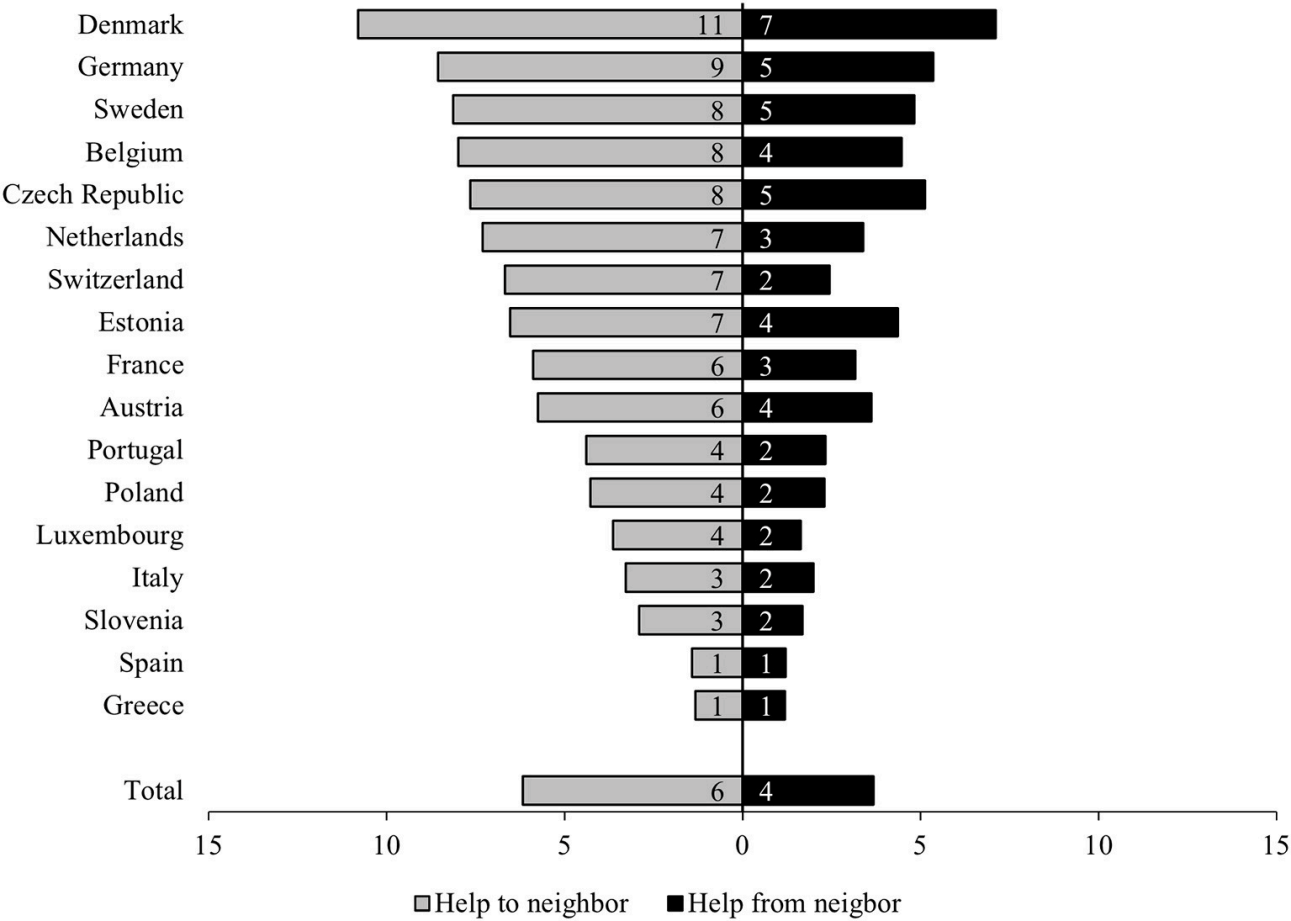
Neighborhood help in Europe. Presented are proportions. Own graph. Data source: Survey of Health, Ageing and Retirement in Europe (SHARE), Waves 1, 2, 4, 5, and 6, Release 6.1.1; *N* = 104,059; own calculations, weighted. Countries sorted in descending order by “help to neighbor”.

Figure 1 illustrates the scope of given neighborhood help and shows that more than 6% of Europeans who participated aged 50 years and older had provided some kind of support to their neighbors within the previous 12 months. Four percent of the respondents reported receiving help from their neighbors.

The results highlighted differences among the included countries. Specifically, the respondents in Denmark, Germany, Sweden, Belgium, and the Czech Republic reported help given to their neighbors more often (8–11%) than others. The situation was similar for received help; respondents from Denmark, Germany, Sweden, the Czech Republic, and Belgium received help from their neighbors quite often (4–7%). In contrast, help among neighbors in both directions (given and receiving) was almost nonexistent (~1%) among those in southern Europe, particularly Greece and Spain. To some degree, these results suggest the existence of an imbalanced gradient of neighborhood help ranging from the north to the south of Europe; however, this pattern was not applicable to all countries, as deviations were observed in the Czech Republic and Luxembourg.

**Figure 2:**
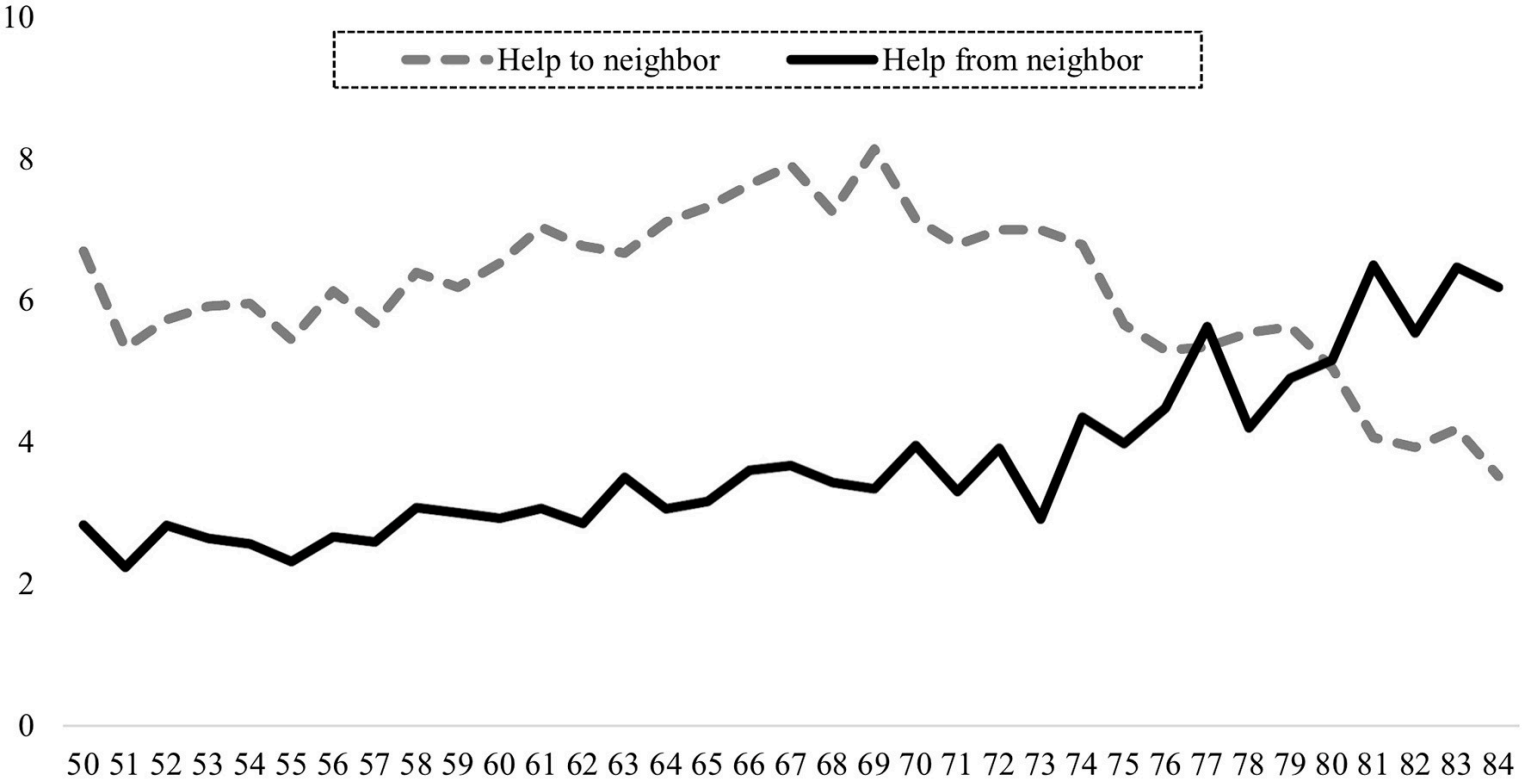
Neighborhood help and life course (proportions). Presented are proportions. Own graph. Data source: Survey of Health, Ageing and Retirement in Europe (SHARE), Waves 1, 2, 4, 5, and 6, Release 6.1.1; *N* = 104,059; own calculations, weighted.

Even when neighbor assistance was reported by participants, the scope varied by age and thus life course. As illustrated in Figure 2, respondents ranging in age from 50 to the early 70s were seen as the main providers (6–8%) of neighborhood assistance. For those of advanced age, i.e., 75 years and above, the help given to a neighbor decreased continually to an average of 2% for those 80 years and older. The provision of neighborhood help followed a reversed U-curve with age; the need for help increased with age, whereas the giving of help was inversely correlated with age. Only 3% of the respondents aged 50 reported receiving support from their neighbors within the previous 12 months; however, the proportion was more than double for those at least 80 years old.

### Determinants of Given and Received Neighborhood Help

To answer the third and fourth research questions, multilevel logistic regression models were developed with consideration for the indicators at the micro-, meso-, and macrolevels (Table 1). The results of the multivariate analyses indicated the presence of reciprocity in neighborhood assistance. Respondents who gave help to their neighbors were also more likely to have received help from a neighbor in the same period, and vice versa. The results also showed that respondents who had provided help within the previous 12 months to someone other than a neighbor had a higher probability of helping their neighbors. Similarly, those who received help from others such as relatives and friends were more likely to receive help from neighbors.

Among microlevel influences, the multivariate analyses confirmed specific life-course patterns. Whereas, the younger respondents had provided more neighborhood assistance, older individuals tended to benefit from the help of at least one neighbor. Gender played a partial role in addition to age and life course; whereas men were more likely to provide help to their neighbors, no gender-specific relationship was found for the receipt of help. Analyses of the influence of socioeconomic status revealed that people with higher education levels were more likely to both provide and receive assistance. Although education is usually positively correlated with wealth, the results indicated the opposite relationship concerning financial resources; respondents in good financial situations with few or no economic restrictions were less likely to either provide or receive help. Migrants were equally likely as natives to support or to be supported by their neighbors.

The results demonstrated that respondents who owned their homes were less likely to provide support for their neighbors but more likely to be the beneficiaries of neighborhood help. In addition, the amount of time that respondents had lived in their current homes affected the likelihood of giving or receiving help. Living in the same home and neighborhood for a lengthy time increased the probability of support among neighbors.

In measuring the impact of life events, the analyses showed that retired individuals were more likely to help their neighbors while being less likely to benefit from such help. The provision and receipt of assistance were significantly influenced by health-based restrictions. Respondents who reported being in good health and having fewer limitations on the instrumental activities of daily life were more likely to provide neighborhood help. Conversely, respondents experiencing fair or poor health and limitations in the ADL and the IADL were more likely to receive assistance from their neighbors.

At the mesolevel, marital status was found to influence neighborhood help, as respondents who had experienced the loss of a partner through death or divorce were less likely to provide help to but were more likely to receive support from their neighbors. Although having a partner did not influence the receipt of neighbor assistance, it decreased the chances of providing it. This was also true for respondents who had at least one parent still alive or one child living nearby, who were less likely to support their neighbors. However, if these respondents were in need and did not have a child living nearby, they could rely on their neighbors.

At the macrolevel, cultural and regional differences within countries were found to influence neighborhood help. The results indicated that the receipt of neighbor assistance was significantly higher outside big cities. Furthermore, the results revealed that the likelihood of older adults' giving or receiving neighborhood help was higher in wealthier countries, as measured by GDP per capita. Stronger countries, measured by social expenditures, had a positive effect on mutual neighbor assistance. Whereas, higher public expenditures for old age did not impact the provision of neighborhood help at the country level, they increased the probability that respondents benefited from such support. A comparatively higher income inequality and societal poverty had lasting and negative effects on neighborhood help, whereas living in countries with a higher population density increased the chances of participation in reciprocal neighbor support. Last, societal satisfaction and wellbeing were reflected in neighborhood assistance; in countries with a high level of life satisfaction, including living situations and social relationships, the provision and receipt of neighborhood assistance were much more common.

## Discussion

This study of older adults living in Europe described the provision and receipt of neighborhood assistance and the predictors of such helping relationships at the micro-, meso- and macrolevels. A major finding was the reciprocal and somewhat substantial helping neighbor interactions reported by the interviewees. However, these mutual help were influenced by personal characteristics and health-related circumstances; social, mainly family-related, resources, and responsibilities; and contextual factors. With these results we can underpin the theoretical work presented in the convoy model of social relations (Antonucci et al., 2010), who conceptionally viewed help among neighbors as affected by personal and contextual variations; therefore, the neighborly assistance is influenced by factors on the micro-, meso-, and macrolevels.

The first research question addressed the incidence of given and received neighborhood help. As only 6% reported having providing help and 4% described having received help across all the countries studied, neighborhood help was clearly not a primary source of social support. Nevertheless, this form of reciprocal intra-neighborhood assistance should not be neglected. Previous studies have indicated that such neighborhood help can positively affect older adults' ability to cope with daily activities (Morita et al., 2010; Cramm et al., 2013; Cain et al., 2018). Even if neighbors do not create strong social ties, it is sometimes easier to spontaneously accept or provide support without strong liabilities (Chen and Feeley, 2014; Aral, 2016). A possible limitation of this survey is that having lived in the same neighborhood for a long time, many interviewees found it difficult to distinguish between friend and neighbor. Lapierre and Keating (2013) demonstrated that the intensity of non-kin care of older people was affected more by the subjective definition of emotional closeness than the binary distinction between “friend” and “neighbor.” Future studies should further investigate the effects of emotional closeness with neighbors and possible accessibility of neighbor assistance on giving and receiving help. Furthermore, future studies should go more into the different forms of help, for example if this help is emotional or instrumental support and if this help also include nursing care.

The second research question, which focused on the reciprocity of help among neighbors, was based on the work of Antonucci and Jackson (1990). Our results showed a high reciprocity among the participants for neighborhood help as well as helping or receiving help from others; herein people who helped their neighbors also helped others and people who received help from others also obtained help from neighbors. Some researchers have proposed that reciprocity is an important element for a neighborhood-based caring community and the provision of help precedes the receipt of help (Greenfield, 2015; Grime, 2018). Reciprocity reflects that older adults were not only the recipients but also the providers of assistance for others in the neighborhood and often filled gaps in services and support (Hand et al., 2018). Furthermore, individuals with active social support networks were more likely to be among those who gave or received help (Dury et al., 2015; Dury, 2018). Social capital theories, such as the work of Putnam (1993) or Bourdieu (1983), imply that norms of generalized reciprocity and voluntary agreements can invigorate social resources that an individual has to maintain everyday life (e.g., Carpiano, 2006; Aldrich and Meyer, 2015; Murayama et al., 2015). Furthermore, offering neighborhood help indirectly affects older adults' everyday competence by engendering a sense of control and mastery (Lang, 2001).

The third research question addressed the role of age in neighborhood help in the European countries that participated. Although neighborhood assistance was reported for all of the older respondents, there were differences among age groups. The multivariate analyses confirmed that more people aged 50 years and older provided rather than received support from neighbors; however, this pattern was not observed for those aged 80 years and older, who were more likely to receive than provide help. The direction of neighborhood help was found to shift during an individual's life course, and changes were associated mainly with the functional limitations and reduced social resources of individuals in advanced age. This result corroborates previous findings that neighborhood and help from neighbors become more important in old age, primarily because of personal limitations (e.g., health, mobility, and social networks) and place attachment (e.g., Lawton and Nahemow, 1973; Cantor, 1979; Shaw, 2005; Wahl et al., 2012; Kaspar et al., 2015). However, the results also indicated that individuals over 80 years of age continued to provide help. Thus, older adults should not be viewed solely as recipients of help in caring communities, but also as providers of help. Accordingly, it is recommended that local social projects that enhance community neighborhood assistance address older individuals to convince them to participate in volunteer work, preferably before their retirement. Such involvement could also improve the social inclusion of older adults in their communities (Scharlach and Lehning, 2013).

Our results highlighted variations across Europe. Respondents from countries such as Denmark, Germany, and Sweden reported more involvement in helping neighbors or receiving their aid than respondents from Spain and Greece. The multivariate analyses showed that country-specific conditions, such as wealth status and overall life satisfaction, predicted given and received neighborhood help (see below).

The last research question addressed differences in given and received neighborhood help in terms of micro-, meso-, and macrolevel influences. Besides highlighting the association between age and neighborhood help, the analyses demonstrated that retirement increased the probability of both giving and receiving neighborhood help. An explanation for these findings is that most older adults spend more time in their neighborhoods after retirement because they are no longer occupied with employment and can therefore spend more time volunteering, such as providing assistance to neighbors. Retired adults are usually older than those who have not yet retired, and to some extent, they are more in need of help in everyday life; thereby the need for neighborhood help arises. (van Tilburg, 1992) found that whereas the support network did not change before and after retirement, post-retirement relationships were evaluated as being more rewarding and the frequency of contact was higher. The data used in this study could not differentiate between frequency of contact or number of neighbors. Therefore, future studies should investigate whether retired individuals can change their social neighbor networks on the basis of the new contacts gained through volunteering.

More male than female respondents reported helping neighbors; however, gender-based differences in receiving neighborhood help were not significant. This result conflicts with previous findings that women were much more likely to provide assistance, particularly emotional support (e.g., Liebler and Sandefur, 2002). It must be emphasized that the current study neither investigated relationships between the number of neighbors and their emotional strength nor examined the intensity of neighborhood assistance; these factors could have a sustainable effect on gender relations.

Respondents with higher levels of education gave and received more help; however, respondents in good financial situations gave and received less help. Whereas the findings on education corroborated previous findings (Seifert, 2016), they conflicted with other results regarding the effects of financial resources (Chen et al., 2016) in that cultural capital was more influential than economic capital. Wealthier older adults could arrange for professional private care or service providers; therefore, they did not rely on their neighbors. Wealthier older adults spent more time outside their own neighborhoods (Mollenkopf et al., 2004), thus resulting in less contact with neighbors.

Respondents who had lived longer in their homes had a higher probability of being among those who gave and received neighborhood help. In a study conducted in a large Swiss city, Seifert (2016) found no significant relationships between time living in a home and the provision of neighborhood assistance; however, the results for the current investigation showed that for both giving and receiving neighborhood help, the time lived in a neighborhood was important for exchanges between neighbors.

Previous studies have indicated that health issues impacting older populations can act as barriers to performing daily activities such as providing neighborhood help (Kaspar et al., 2015; WHO, 2015), and our analyses confirmed these findings. Respondents who reported overall poorer health and greater limitations in daily activities were more likely to be recipients of help from neighbors. The results showed that health status and health-related limitations also influenced the provision of help; older adults who were in good health were better able to help their neighbors.

The influence of social context was explored in addition to personal characteristics, and the results showed that older adults who had experienced the loss of a partner were less likely to provide neighborhood help and more likely to receive support. Whereas, having a partner did not significantly impact receiving help from neighbors, it decreased the chances of helping them, which suggests that respondents who lived with partners might have prioritized support for their closest and most intimate relations. The analyses showed that respondents with at least one parent still alive or one child living nearby were less likely to be among those who provided help to their neighbors. If respondents needed support but did not have a child living nearby, their neighbors were more likely to support them with time-related help. Caring for a parent or child can be time-consuming (Brandt et al., 2009), thus reducing the time available for providing neighborhood help. However, children living nearby can be also a resource for individuals of advanced age (Deindl and Brandt, 2017).

In addition to individual and family variables, structural circumstances were found to be important determinants of giving or receiving neighborhood help. The results identified a gap between the large cities and the less urban regions in Europe regarding the receipt of neighborhood help, but not its provision. In large cities, the probability of receiving help from neighbors was lower, which was consistent with the results of previous studies (Amato, 1993). Interestingly, no significant difference was found regarding the provision of help, which indicates that the provision of assistance occurred everywhere regardless of regional background. Levasseur et al. (2015) did not initially find differences in social participation across locations; however, they were able to identify differences after controlling for social and infrastructural neighborhood resources. Therefore, the living area had less effect on the social networks of neighborhood participation than did specific neighborhood and personal characteristics, such as openness to participation.

By focusing on various European countries, the study highlighted correlations between country-specific economic wealth, less social inequality, public social expenditures, and life satisfaction on the one hand and the willingness to help neighbors and to ask neighbors for help on the other. Therefore, it can be concluded that country-specific characteristics operate as a resource for social exchange with a neighborhood.

Future studies should investigate the possibilities for neighborhood participation for older adults who do not provide or receive neighborhood help. Grime (2018) found that older adults were often reluctant to ask for help; therefore, it is important that neighborhood contacts should be promoted by local social institutions and policy makers. Nevertheless, Nocon and Pearson (2000) warned policy-makers and practitioners against overreliance on the assistance from neighbors, which cannot replace professional social support; accordingly, it is important to combine professional care with (existing) neighborly assistance (Pleschberger and Wosko, 2017).

## Limitations

Because the present study focused on 17 European countries, caution must be used in generalizing the findings. The dependent variables were binary; therefore, not all possible facets of neighborhood help (e.g., frequency, emotional attachment, and target persons) could be considered. A detailed examination of the relationships (e.g., their emotional closeness, the types of help, and the intensity of the contact and help) among neighbors and the history of those relationships was not possible, as these specific data were not collected by SHARE. Respondents were not asked about their general contact with neighbors but rather were asked about their social support networks in just two waves and were allowed to identify specific persons, who tended to be mostly family and friends. Consequently, a neighbor named by a respondent denoted some form of meaningful relationship. More granular network analyses and longitudinal data that include the life-course developments and different form of relationships among neighbors and the outcomes of these relationships are required to identify the underlying effects of daily neighboring among the older population. According to our study and the data, we only could partly address all aspects of neighboring among older adults, so we focused on the determinants of mutual neighborly assistance on the micro-, meso-, and macrolevel.

## Conclusion

For the European countries considered in this study, the findings provide support for the importance of the living environment in old age and for the existence of lively neighborhoods with social contact and reciprocal neighborhood help for this population. However, this neighborly assistance varies between countries, areas, and neighborhood units. Based on empirical findings, some practical implications arise. First, as an overall theoretical-based finding, it is recommended to look not only at the person and their functional ability but also at the relationship between the individual and the context in which the individual lives. According to the results of the present study, the provision and receipt of neighborhood help are driven by personal characteristics, living situations, social resources, and responsibilities as well as contextual factors. Therefore, not only should researchers add contextual information to their research (as does here), but also practical gerontological social worker working with older adults. Second, this context-based approach suggests that age-friendly environments help foster social contact in a neighborhood (Jeste et al., 2016); as such, cities and communities should invest financial resources to provide neighborhoods that are physically accessible for older adults, enable public meeting spaces, and encourage neighboring by financial assistance of neighborhood assistances, who provide a structure of coordination neighborhood help by connection neighbors who need help with neighbors who can provide it. Moreover, interventions to promote the social exchange in a neighborhood should focus on the individual's life course, social networks, and country characteristics. Third, the study showed a high reciprocity of neighboring among older adults. However, reciprocity also implies that older adults should not be viewed only as recipients but also as active providers of assistance to others in the neighborhood. Indeed, active helpers often filled gaps in social services in the neighborhood (Hand et al., 2018). Being active gives older adults also a feeling of meaningful social involvement and affects thereby the general social cohesion in a neighborhood (Joe et al., 2019). Therefore, older people should not have to feel like “demanders,” and their neighbors should not have to be only “helpers.” Fourth, the results of the presented study produced a basic assumption “help needs contact”; this means that asking neighbors for help or providing help to neighbors starts with contacting. Though seemingly basic, it is a critical task that indicates that community social services seeking to build an informal social support network in the neighborhood should start by developing a structure of contacts to bring neighbors together. The initial contact between neighbors is key as neighbors often do not know each other or the last time intensive contact occurred was years ago; therefore, contacts must be initiated. This is especially important considering research findings that not every older adult has someone to connect with and, consequently, someone to ask for help (Bunt et al., 2017). To initiate contact with neighbors, opportunities to meet neighbors are important, such as thru regular events or activities in a neighborhood provided for example by neighborhood assistances, churches, or senior citizens' organizations. Therefore, regional interventions to encourage social support among neighbors should provide opportunities for neighbors to meet one another in and outside residences.

## Author Contributions

All authors listed have made a substantial, direct and intellectual contribution to the work, and approved it for publication.

### Conflict of Interest Statement

The authors declare that the research was conducted in the absence of any commercial or financial relationships that could be construed as a potential conflict of interest.
